# Effect of flaxseed supplementation and exercise training on lipid profile, oxidative stress and inflammation in rats with myocardial ischemia

**DOI:** 10.1186/1476-511X-11-129

**Published:** 2012-10-05

**Authors:** Howaida A Nounou, Maha M Deif, Manal A Shalaby

**Affiliations:** 1 Department of Medical Biochemistry, Faculty of Medicine, Alexandria University, Alexandria, 21111, Egypt; 2 Current address: Department of Biochemistry, College of Science, King Saud University, Riyadh, 11495, Saudi Arabia; 3 Department of Physiology, Faculty of Medicine, Alexandria University, Alexandria, 21111, Egypt; 4 Genetic Engineering and Biotechnology Research Institute (GEBRI), City for Scientific Research and Technology Applications, Alexandria, Egypt

**Keywords:** Flaxseed, Exercise, Ischemia, Pentraxin, Troponin, Cytokines, Paraoxonase, Lipid profile

## Abstract

**Background:**

Flaxseed has recently gained attention in the area of cardiovascular disease primarily because of its rich contents of α-linolenic acid (ALA), lignans, and fiber. Although the benefits of exercise on any single risk factor are unquestionable, the effect of exercise on overall cardiovascular risk, when combined with other lifestyle modifications such as proper nutrition, can be dramatic.

This study was carried out to evaluate the protective role of flaxseed and exercise on cardiac markers, lipids profile and inflammatory markers in isoproterenol (ISO)-induced myocardial ischemia in rats.

**Methods:**

The research was conducted on 40 male albino rats, divided into 4 groups (n=10): group I served as control, group II has acute myocardial ischemia induced by isoproterenol, groups III and IV have acute myocardial ischemia induced by isoproterenol pretreated with flaxseed supplementation orally for 6 weeks, additionally group IV practiced muscular exercise through swimming.

**Results:**

Alterations of lipid profile, cardiac and inflammatory markers (Il-1β, PTX 3 and TNF- α) were observed in myocardial ischemia group. Flaxseed supplementation combined with exercise training showed significant increase of HDL and PON 1, on the other hand cardiac troponin, Il- 1β and TNF- α levels significantly decreased as compared to myocardial ischemic group. Receiver Operating Characteristics (ROC) analysis of cTnI, PTX 3, Il-1β and TNF- α revealed a satisfactory level of sensitivity and specificity.

**Conclusion:**

Regular exercise enhances the improvement in plasma lipoprotein levels and cardiovascular protection that results from flaxseed supplementation by mitigating the pathophysiology of atherosclerosis. Elevation of HDL, the antioxidant PON 1 and the cardioprotective marker PTX 3 emphasizes the protective effects of flaxseed and muscular exercise mutually against the harmful effects of acute myocardial ischemia.

## Background

Coronary heart disease (CHD) is the single largest cause of death in the developed countries and is one of the leading causes of disease burden in developing countries as well. In 2001, there were 7.3 million deaths and 58 million disability adjusted life years (DALYs) lost due to CHD worldwide [1,2]. Reduction of heart oxygen supply due to coronary obstruction leads to diminution of oxygen supply to the mitochondria to support oxidative phosphorylation and eventually ischemia [3]. Dietary interventions, have recently received attention as effective strategies to prevent, mitigate, or reduce risk factors of some diseases [4]. Considerable cardiovascular disease researches focus on the cardioprotective effects of fish oils and of individual n-3 PUFA [5,6]. Experimental evidence suggests that regular consumption of (n-3) PUFA is particularly effective in protecting against the damaging effects of myocardial ischemia [7]. PUFA such as linoleic acid and alpha-linolenic acid (ALA) cannot be synthesized in the human body; therefore they must be obtained from the diet and thus are called essential fatty acids. Common sources of n-3 PUFA include fish oils (eicosapentaenoic acid and docosahexaenoic acid) and some plant oils such as canola and flaxseed oils (ALA). Flaxseed is a rich source of PUFAS and contains an average of 41% oil. Some varieties contain 45–60% n- 3 fatty acid [ALA; 18:3 (n-3)], which has been reported to increase the cellular concentrations of eicosapentaenoic acid (EPA) and docosahexaenoic acid (DHA) in both platelets and blood vessels [8]. In a case–control study of 96 middle-aged men with incident stroke, Simon et al. [9] found that a significant 0.06% increase in phospholipid ALA content was associated with a 28% decrease in the risk of stroke [10].

The effects of flaxseed supplementation on plasma lipids have remained controversial. Some reports recorded that flaxseed (12.5 g/day) prevented the rise in plasma triglyceride levels over a 16-week feeding period in a hypercholesterolemic rabbit model but had no effect on triglycerides levels in low density lipoprotein receptor (LDLr)−/− mice supplemented with 0.04 g/day of flaxseed during 24 weeks. Others reported that dietary flaxseed had no effect on cholesterol levels in rabbits, but even low doses (0.04– 0.2 g/day) of dietary flaxseed significantly reduce plasma cholesterol levels in the LDLr−/− mice [11,12].

Cardiac troponin (cTnI) has been established as one of the most useful biomarkers for risk assessment and management of suspected acute coronary syndrome patients. Previous studies reported that elevated cTnI levels are highly specific for cardiac injuries [13-15].

Exercise training confers sustainable protection against myocardial infarction in animal models and has been associated with improved survival following a heart attack in humans. It is still not clear the mechanism by which exercise training protects the heart, but some studies have suggested that it alters a number of classical signaling molecules [1]. To gain insight about the cardiovascular effects of flaxseed and to understand how this substance works in our bodies, our experimental studies have examined the prophylactic role of flaxseed supplementation alone and in combination with exercise in isoproterenol-induced myocardial ischemia in rats.

## Results and discussion

Many studies have focused on improving lipid profiles (as one of the most important risk factors of chronic diseases) by planning a better diet or introducing herbal treatments. Lipid profile improving effect of flaxseed has long been studied and many literatures have related its effect to high fiber, ALA and lignans content of flaxseed [16].

**Figure 1:**
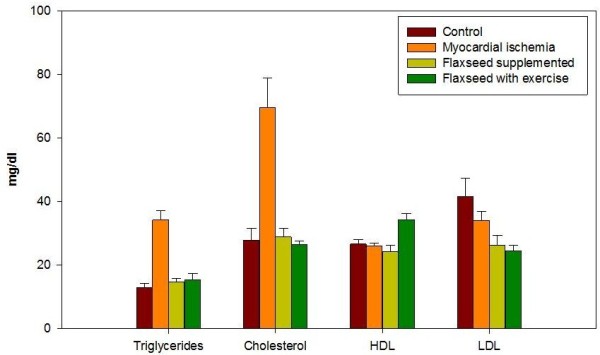
**Triglycerides, cholesterol, HDL and LDL levels measured in plasma of the different studied rat groups.** Triglycerides and cholesterol levels increased after ischemia in rat’s plasma. Treatment with flaxseed separately and in combination with exercise attenuated the increase in triglycerides and cholesterol levels. HDL slightly decreased after ischemia in plasma of untreated rats and flaxseed treated rats. Combined treatment with flaxseed and exercise markedly increased HDL level in rat’s plasma. LDL slightly decreased after ischemia in plasma. Treatment with flaxseed separately and in combination with exercise markedly decreased LDL levels in rat’s plasma. Data from plasma of ten rats in each group are shown as mean ± S.D.

The comparison of blood lipid profiles of control and studied groups after treatment period and ischemia revealed that plasma triglyceride levels in the myocardial ischemia group II was 34.2±3.0 mg/dl with increase up to 263.1% relative to control (Table 1 and Figure 1). Elevated triglyceride levels in group II the myocardial ischemia group could be due to decreased activity of lipoprotein lipase in acute myocardial ischemia [17]. Alterations in the lipid profiles of isoproterenol-treated rats, attributed to enhanced lipid biosynthesis by cardiac cyclic adenine monophosphate (cAMP) may be responsible for the triglyceride elevation in group II [18].

**Table 1:** Levels of the cholesterol, triglyceride, LDL and HDL in plasma of control and experimental groups of rats

**Lipid profile**	**Groups**	**Median**	**Interquartile Range**	**Mean**
**Triglycerides (mg/dl)**	(1) Control	12.8	12.2-13.8	13.0
	(2) Cardiac ischemia	34.5	32.8-36.8	34.2 ^a^***
	(3) Flaxseed supplemented	14.7	13.8-15.5	14.6 ^b^*^, d^***
	(4) Flaxseed with exercise	14.9	13.9-15.6	15.2^c^**^,E^***
**Cholesterol (mg/dl)**	(1) Control	26.7	25.4-29.6	27.9±3.6
	(2) Cardiac ischemia	74.4	58.6-75.4	69.6^a^***
	(3) Flaxseed supplemented	28.0	26.8-30.8	28.8 ^d^***
	(4) Flaxseed with exercise	26.5	26.0-27.0	26.4 ^E^***
**HDL (mg/dl)**	(1) Control	26.8	25.4-27.6	26.5
	(2) Cardiac ischemia	25.9	25.2-26.7	25.9
	(3) Flaxseed supplemented	24.0	22.3-26.0	24.2^b^*
	(4) Flaxseed with exercise	34.3	32.8-36.2	34.3^c^***, ^E^***^, F^***
**LDL (mg/dl)**	(1) Control	43.3	33.7-46.0	41.5
	(2) Cardiac ischemia	34.3	31.5-36.4	33.9 ^a^*
	(3) Flaxseed supplemented	27.4	24.1-28.9	26.3^b^***^, d^***
	(4) Flaxseed with exercise	24.4	23.0-26.3	24.5^c^***, ^E^***^,^

a: Significant difference between control and myocardial ischemia. b: Significant difference between control and flaxseed supplemented rats. c: Significant difference between control and flaxseed with exercise group. d: Significant difference between groups 2,3. E: Significant difference between groups 2,4 F: Significant difference between groups 3,4.

*: P<0.05; **: P<0.001; ***: P<0.0001.

Flaxseed supplementation in group III has increased plasma triglycerides to 14.6±1.2 mg/dl with increase up to 112.3% relative to control (P <0.05). Flaxseed supplementation combined with exercise training has increased triglycerides to 15.2±2.1 mg/dl with increase up to 116.9% as compared to the control group (P <0.001) (Table 1 and Figure 1). Previous studies reported that elevated triglyceride levels may depend on nutritional basis which could explain the elevated level of triglycerides in groups III and IV which received flaxseed supplementation [19].

A remarkable elevation of cholesterol level to 69.6 ±9.2 mg/dl with increase up to 249.5% relative to control (P <0.0001) was determined and attributed to myocardial ischemia in group II. Flaxseed supplementation in group III dropped cholesterol level to nearly the control level 28.8±2.8 mg/dl with mild increase of 103% compared to control. Cholesterol level decreased significantly due to exercise training combined with flaxseed in group IV to 26.4±1.0 mg/dl compared to myocardial ischemia group (P <0.0001) with decrease down to 94.6% relative to control (Table 1 and Figure 1).

The myocardial ischemia group II has LDL level of 33.9±2.8 mg/dl, which is significantly higher than group III (flaxseed supplemented group and group) IV (flaxseed supplemented group combined with exercise training) (P <0.0001). Flaxseed supplementation in group III decreased LDL to 26.3±3.0 mg/dl with decrease down to 63.4% relative to control, while exercise training combined with flaxseed supplementation decreased LDL to 24.5±1.8mg/dl with decrease down to 59% in group IV as compared to the control (Table 1 and Figure 1). The reported decrease of total cholesterol and LDL in group III and group IV could be due to the protective effects of lignan complex isolated from flaxseed in reducing the extent of hypercholesterolemic atherosclerosis [20].

On the other hand, there was a significant increase of high density lipoprotein (HDL) to 34.3±1.9 mg/dl in group IV which was on flaxseed supplementation and exercise training as compared with the other groups (P <0.0001), with increase up to 129.4% increase as compared to the control (Table 1 and Figure 1). Flaxseed supplementation in group III was ineffective towards producing alterations in plasma HDL which is 24.2±1.9 mg/dl with decrease down to 91.3% compared to control (Table 1 and Figure 1). Most previous studies reported no changes in HDL levels in response to dietary flaxseed [11]. The significant increase in HDL due to flaxseed supplementation and exercise may be explained by stimulation of lipid oxidation during activity and in post exercise recovery. Alterations in the transport of blood lipids, with a higher ratio of HDL to LDL increased lipoprotein lipase activity, which increases the use of circulating triglycerides as fuel and increases their clearance. Activation of this enzyme also speeds up the conversion of the very low density lipoproteins (VLDL) to HDL [21,22].

**Figure 2:**
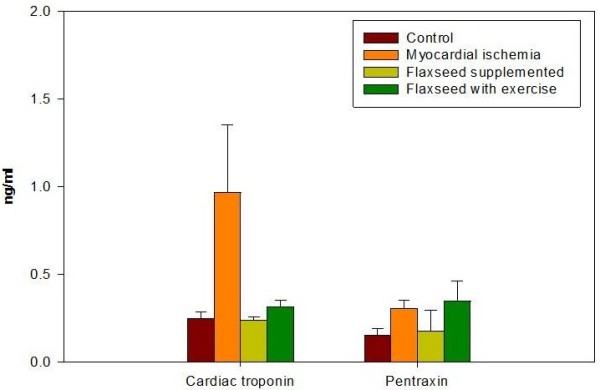
**cTnI and PTX 3 levels measured in plasma of the different studied rat groups.** cTnI markedly increased after ischemia in plasma. Treatment with flaxseed separately and in combination with exercise attenuated the increase in cTnI levels. PTX 3 levels increased after ischemia in plasma. Treatment with flaxseed attenuated the increase in PTX 3 level. Combined treatment with flaxseed and exercise increased PTX 3 level in rat’s plasma. Data from plasma of ten rats in each group are shown as mean ± S.D.

While the marker of ischemic myocardial injury cTnI has increased due to myocardial ischemia in group II to 0.97±0.4 ng/ml with increase up to 485% as compared to the control, flaxseed supplementation in group III ameliorated the level of cTnI to 0.2±0.02 ng/ml (P < 0.0001) almost 100% as the control. Flaxseed supplementation combined with training in group IV increased cTnI to 0.3±0.04 ng/ml with elevation up to 150% as compared to control as shown in Table 2 and Figure 2. The antiatherogenic potential benefits of flaxseed, that has been received in both group III and IV, protected against the loss of endothelial-dependent vascular relaxation in cholesterol fed animals [23]. The protective effect of flaxseed has been deteriorated in group IV by the viable release of myocytes cTnI through stretch- related mechanism mediated by integrins [24].

**Table 2:** Levels of cardiac troponin and paraoxonase 1 in plasma of control and experimental groups of rats

**Cardiac markers**	**Groups**	**Median (IQR)**	**Interquartile Range**	**Mean**
**Cardiac troponin (ng/ml)**	(1) Control	0.2	0.2-0.3	0.2
	(2) Cardiac ischemia	0.8	0.7-1.0	0.97 ^a^***
	(3) Flaxseed supplemented	0.2	0.2-0.2	0.2 ^d^***
	(4) Flaxseed with exercise	0.3	0.3-0.4	0.3^c^**, ^E^***^, F^***
**Paraoxonase 1 (ng/ml)**	(1) Control	92.6	89.6-94.7	92.3
	(2) Cardiac ischemia	83.4	82.6-85.4	83.6 ^a^***
	(3) Flaxseed supplemented	77.5	73.5-79.0	75.9 ^b^***^, d^****
	(4) Flaxseed with exercise	99.0	97.0-101.3	99.0 ^c^***, ^E^***^, F^***

a: Significant difference between control and myocardial ischemia. b: Significant difference between control and flaxseed supplemented rats. c: Significant difference between control and flaxseed with exercise group. d: Significant difference between groups 2,3. E: Significant difference between groups 2,4 F: Significant difference between groups 3,4.

*: P<0.05; **: P<0.001; ***: P<0.0001.

**Figure 3:**
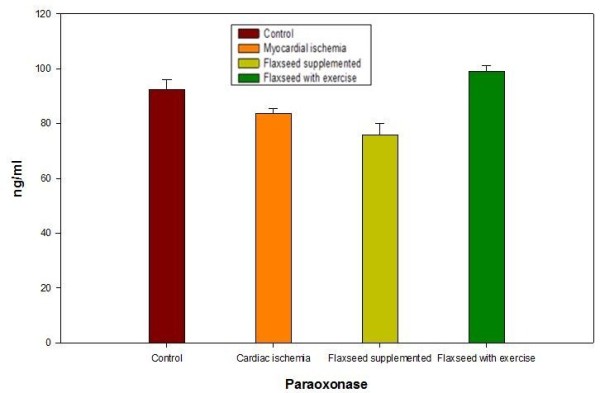
**PON 1 levels measured in plasma of the different studied rat groups.** PON 1 decreased after ischemia in plasma of untreated group and flaxseed treated rats. Treatment with flaxseed in combination with exercise increased PON 1 level in rat’s plasma. Data from plasma of ten rats in each group are shown as mean ± S.D.

While Paraoxonase (PON 1), HDL associated enzyme, is capable of inhibiting LDL oxidation, susceptibility to coronary artery disease has shown to be associated with polymorphisms of PON gene (PON 1) in different population [14]. The antioxidant activity of HDL is believed to reside in its associated enzymes, particularly PON 1. To evaluate this correlation we measured PON 1 in all the studied groups. Data in Table 2 and Figure 3 showed a significant decrease in PON 1 in myocardial ischemia level to 83.6±2.0 ng/ml with decrease to 90.6% relative to control (P < 0.0001). Plasma PON 1 levels significantly decreased to 75.9±4.1 ng/ml with a decrease to 82.2% relative to the control due to flaxseed supplementations in group III (P < 0.0001). The significant increase of PON 1 in group IV to 99.0±2.2 ng/ml compared with other groups (P < 0.0001) with increase to 107.3% compared to control may be due to the training exercise associated with flaxseed supplementation in this group. This could be explained by the mutuality with HDL antioxidative capacity and the moderate intensity exercise training [25,26].

**Figure 4:**
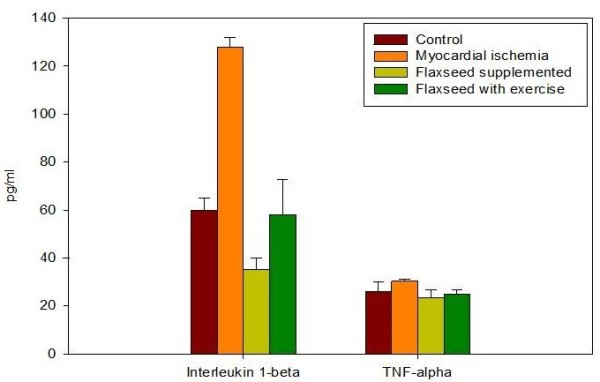
**IL-1β and TNF-α levels measured in plasma of the different studied rat groups.** IL-1β increased markedly after ischemia in plasma. Treatment with flaxseed separately and in combination with exercise attenuated the increase in IL-1β levels. TNF-α levels increased after ischemia in plasma. Treatment with flaxseed separately and in combination with exercise attenuated the increase in TNF-α level. Data from plasma of ten rats in each group are shown as mean ± S.D.

Interleukin- 1β (IL-1β) levels, showed a significant increase in group II (the myocardial ischemia group) up to 127.8±4.0 pg/ml with increase up to 213% relative to the control group control group (P < 0.0001) (Table 3 and Figure 4). This could be due to the proinflammatory role of IL-1β in ischemic diseases [27]. IL-1β stimulates inflammatory cells like leukocytes, and induces systemic inflammatory reactions like the expression of acute-phase proteins in the liver. Leukocyte IL-1β expression is induced in the ischemic and reperfused heart [28]. Previous studies reported that flaxseed exerts anti-inflammatory actions and antioxidant potential [29] and that the important antiatherogenic role of ALA may involve a potent anti-inflammatory role [10]. This could explain the significant decrease in plasma IL-1β levels in group III which received flaxseed supplementation to 35.1±4.8 pg/ml as compared with the myocardial ischemia group II (P <0.0001) with decrease down to 58.5% relative to the control (Table 3 and Figure 4).

**Table 3:** Levels of the inflammatory markers IL-1β, PTX 3 and TNF-α in plasma of control and experimental groups of rats

**Inflammatory markers**	**Groups**	**Median**	**Interquartile Range**	**Mean**
**Interleukin 1-beta (pg/ml)**	(4) control	59.5	57.0-60.3	60.0
	(2) Cardiac ischemia	128.0	125.5-130.8	127.8 ^a^***
	(3) Flaxseed supplemented	34.7	33.0-40.0	35.1 ^b^***^, d^***
	(4) Flaxseed with exercise	61.5	47.3-68.0	58.1 ^E^***^, F^***
**Pentraxin 3 (ng/ml)**	(1) Control	0.16	0.13-0.18	0.15
	(2) Cardiac ischemia	0.32	0.25-0.35	0.3^a^***
	(3) Flaxseed supplemented	0.15	0.12-0.18	0.18
	(4) Flaxseed with exercise	0.38	0.24-0.41	0.35 ^c^**^, F^***
**TNF-alpha (pg/ml)**	(1) Control	26.6	20.7-30.2	25.9
	(2) Cardiac ischemia	30.7	29.3-31.0	30.3^a^*
	(3) Flaxseed supplemented	22.4	21.2-24.9	23.5 ^d^***
	(4) Flaxseed with exercise	25.0	23.7-26.5	24.9 ^E^***

a: Significant difference between control and myocardial ischemia. b: Significant difference between control and flaxseed supplemented rats. c: Significant difference between control and flaxseed with exercise group. d: Significant difference between groups 2,3. E: Significant difference between groups 2,4 F: Significant difference between groups 3,4.

*: P<0.05; **: P<0.001; ***: P<0.0001.

IL-1β has significantly increased in group IV which was on combined flaxseed supplementation and exercise training as compared to group III on flaxseed supplementations (P <0.0001). The upturn in IL-1β in group IV reached 58.1±14.4 pg/ml with decrease down to 96.8% compared to the control group (Table 3 and Figure 4). Moldoveanu et al. 2000; reported that exercise induces an elevation in circulating inflammatory markers, which may be due to metabolic events and immune function including increased glucose use, lactate accumulation and leukocyte counts [30]. The type of physical activity, as well as the intensity and duration of the exercise has been reported to affect the cytokine profile [31]. The mechanism for inhibition of the tumor necrosis factor (TNF-α) and IL-1β synthesis by dietary n-3 fatty acids is unknown, but may be explained by the fact that n-6 eicosanoid synthesis is inhibited by n-3 fats, and n-6 eicosanoids can affect cytokine synthesis. Leukotriene B4 is a possible mediator because its synthesis can be inhibited by n-3 fatty acids and it is reported to be a simulator of TNF-α and IL-1β production [32].

TNF-α is a prominent element in the cytokine hypothesis of heart disease and has been the focus of research in myocardial ischemia [2]. Table 3 and Figure 4 showed a significant increase of plasma TNF-α in the myocardial ischemia group II to 30.3±0.9 pg/ml with increase up to 117% as compared to the control group P < 0.05. These results are in agreement with previous work stating that over-production of TNF-α contributes to cardiac dysfunction through increase of TNF-α gene expression and peptide synthesis in the myocardium that are associated with NF-kappaB activation [33]. There was diminution in plasma TNF- α to 23.5±3.2 pg/ml with decrease down to 90.7% change in group III as compared to the control group, this is in accordance with previous data demonstrating that flaxseed consumption inhibits TNF-α synthesis and reduces cytokine production by peripheral mononuclear cells [34]. Group IV which received flaxseed supplementation and exercise training showed plasma levels of TNF-α 24.9±2.0 pg/ml with decrease down to 96.1% as compared to control group (Table 3 and Figure 4).

There was a significant decrease in TNF-α levels in groups III and IV as compared to the myocardial ischemia group (P < 0.0001) (Table 3 and Figure 4). Three studies reported anti-inflammatory effects of flaxseed in healthy adults after 4 to 12 weeks of dietary intervention, including reductions in inflammatory markers, such as TNF- α, IL-1β, thromboxane B5, prostaglandin E5, soluble vascular cell adhesion molecule-1 (sVCAM-1), soluble E-selectin, and serum C- reactive protein (CRP) levels [35-38]. However, the majority of clinical dietary intervention trials investigating the effects flaxseed on markers of inflammation have reported no effect on serum levels of IL-6, TNF-α, soluble intercellular cell adhesion molecule-1, sVCAM-1, monocyte chemoattractant protein-1, CRP, or serum amyloid A (SAA) protein [39,40].

Recent research reported that moderate endurance activity influenced circulating cytokine levels specially the TNF- α levels which could be due to production of IL-6 produced by exercising muscle as an anti-inflammatory effect [41].

Pentraxin 3 (PTX 3) has been proposed as candidate marker for acute and chronic heart diseases [12]. PTX3 synthesis is stimulated in endothelial cells, macrophages, myeloid cells, and dendritic cells by cytokines and endotoxins such as bacterial products, interleukin-1, and TNF-α [10-13]. Our results showed a significant increase in PTX 3 levels in myocardial ischemia group II to 0.3±0.05 ng/ml with increase up to 200% relative to control ( P < 0.0001) (Table 3 and Figure 2). This has been in correlation with the elevated levels of tissue mRNA expression and circulating PTX 3 reported in a model of acute myocardial infarction which was explained by the role played by interleukin-1R-MyD88 in inducing PTX 3 transcript after ischemia. [12,41,42]. A significant increase of plasma PTX 3 was observed in group IV that were receiving flaxseed supplementation and on exercise (0.35±0.11ng/ml) as compared to group I the control group (0.15±0.04 ng/ml) and to group III that was on flaxseed supplementation only (0.18±0.12 ng/ml) (P< 0.001, P< 0.001 respectively). The increment in PTX 3 levels due to flaxseed supplementation and exercise was up to 233.3% compared to the control group (Table 3 and Figure 2).

The elevated levels of PTX 3 in group IV is in concurrence with the increased levels of HDL in the same group as compared with sedentary controls. Plasma PTX 3 levels increases with cardiovascular disease, suggesting that plasma PTX 3 levels may increase in order to confer protection against cardiac tissue damage [43]. The importance of pentraxin in protection against cardiovascular diseases has been established in the PTX 3-knockout mice which acquired exacerbated heart damage and increased inflammatory response [43]. Data obtained in animal models of CVD suggest that, pentraxin might play a marked protective response to severe cardiac injury more than a role in the pathogenesis of damage [44]. These findings would suggest that PTX 3 could be either a bystander resulting from the action of proinflammatory cytokines in damaged tissues or a molecule directly involved in the extent and outcome of the inflammatory response.

Our results showed alterations in the lipid profiles of isoproterenol-treated rats (group II); with increase of total cholesterol and triglycerides, the marker of ischemic myocardial injury, cTnI also increased in the same group, all the inflammatory biomarkers IL-1β, PTX 3 and TNF- α level increased with myocardial ischemia.

Flaxseed supplementation in group III has returned the levels of total cholesterol and triglycerides to almost the same levels as the control while LDL decreased far below the control. The marker of ischemic myocardial injury cTnI also returned to same level as control. The inflammatory biomarkers, PTX 3 and TNF-α decreased to almost same level as the control, while IL- 1β decreased to a level much below the control level.

Flaxseed supplementation combined with muscular exercise had the same improving effect as flaxseed except for the significant increase of HDL which was considered as highly beneficial; the cardiac marker paraoxonase also increased which would be expected with exercise. We have also identified a slight rise of the inflammatory biomarker TNF- α and a significant increase of IL-1β as compared to flaxseed supplementation only. There was also a significant increase of the cardioprotective marker pentraxin in this group.

Receiver Operating Characteristics (ROC) analysis presented in Table 4, supports the previous discussion and suggestions which are based on the obtained data. cTnI, PTX 3, IL-1 β and TNF- α values recorded area under the curve (AUC) near 1 and satisfactory levels of specificity and sensitivity and hence they could be used as biochemical markers for detection of protective effects of flaxseed and muscular exercise.

**Table 4:** Receiver-operating (ROC) characteristic analysis of the predictive value of cardiac and inflammatory markers

**Parameter**	**Area under the curve**	**Best cutoff value**	**Sensitivity%**	**Specificity%**
Cardiac troponin	1.0	0.515	100	100
Paraoxonase 1	0.0	--	--	--
Interleukin-1β	1.0	96.5	100	100
Pentraxin 3	1.0	0.22	100	100
TNF-α	0.83	28.5	100	70

Analysis of cardiac troponin, paraoxonase 1, interleukin -1β, pentraxin 3 and TNF- α in myocardial ischemia rats and their controls (n=20). All the tested parameters except for paraoxonase 1 have area under the curve (AUC) near 1 and appropriate levels of specificity and sensitivity.

## Conclusion

This study highlights that combination of flaxseed and exercise is one of the promising cytoprotective elements for improving defense mechanisms in the physiological systems against oxidative stress and inflammation caused by myocardial ischemia.

## Methods

The study was conducted on 40 adult male albino rats, of body weight 150–200 g obtained from Animal Care Centre, Alexandria University, Egypt. The animals were kept in a 12-hour light/dark cycle, at a temperature of 22°C ± 2°C with relative humidity 50% ± 20%, and ventilated with filtered non-recycled air. They were fed on standard chow pellets (Gebze Food Factory, Kocaeli, Turkey) and tap water *ad libitum* for the entire test period. The experimental procedures were carried out according to the National Institute of Health Guidelines for Animal Care and approved by the Local Ethics Committee, Alexandria University.

### Chemicals and reagents

Flaxseed was purchased from General Nutrition Corporation; Pittsburgh, PA 15222 USA. The components of flaxseed were as following typical fatty acid profile per serving; alpha-linolenic acid (ALA) (Omega-3); 7700 mg, linoleic acid (LA)(Omega-6); 2170 mg, oleic acid (OA)(Omega-9); 2240 mg. Amount per serving; calories; 130.00, calories from fat; 130.00, total fat; 14.00 g, saturated fat; 1.50 g, trans fat; 0.00 g, polyunsaturated fat; 10.00 g, monounsaturated fat; 2.50 g; cholesterol; 0.00 mg, sodium 0.00 mg, total carbohydrate 0.00 g, protein 0.00 g. Isoproterenol was purchased from Yangzhou O & L Chemical Industry **Co**., Ltd. All other chemicals used in this study were of high analytical grade. Kits for cholesterol, triglyceride and HDL-cholesterol were purchased from Randox Laboratories Ltd. (CRUMLIN, CO. Antrim, UK). Kits for high sensitivity cTnI was purchased from Life Diagnostics, Inc., TNF-α and IL-1β kits were purchased Quantikine Immunoassay kits (R&D Systems, Inc, USA), PTX 3 kit was from CUSABIO BIOTECH Co., Ltd. and PON-1 kit was purchased from Life Science Co.

### Study design

Rats were divided into 4 groups (n=10):

Group I: served as a control group, treated orally with saline for 6 weeks and then injected subcutaneously with saline for 2 consecutive days.

Group II: was treated with isoproterenol subcutaneously (85mg/kg) for 2 consecutive days [45] for induction of myocardial ischemia.

Group III: received flaxseed oil orally by gavage tube in a dose of 0.4 g/day for six weeks then myocardial ischemia was induced by isoproterenol as described in group II [11,12].

Group IV: was trained with exercise preconditioning in the form of mere swimming. The swimming exercise was performed in a 120 cm deep x 80 cm wide cylindrical tank, with water temperature of 31 ± 1°C. After one week of acclimatization, the swimming protocol was started. Swimming was performed for 6 weeks, 5 days per week and one hour per day [46], in addition, this group (IV) was treated with flaxseed supplementation as group III, and then myocardial ischemia was induced after two days of relaxation from exercise by isoproterenol in same way as group II.

At the end of the experimental period,12 hours fasting after the second dose of isoproterenol injection, rats were sacrificed by decapitation under ether anaesthesia and blood was collected in EDTA containing tubes. After the samples were collected, all sample tubes were centrifuged at 1500 rpm for 10 min at 4 °C to separate plasma. Plasma lipids were measured. The remaining part of the plasma was immediately aliquoted into eppendorf tubes placed on ice and immediately stored at −80 °C until ELISA measurements of high sensitivity cTnI, TNF-α, IL-1β, pentraxin-3 and paraoxonase-1 were performed.

### Assessment of lipid profile

Total cholesterol was measured by enzymatic colorimetric cholesterol oxidase and peroxidase method [47], HDL by phosphotungstic acid, magnesium ions precipitate LDL and VLDL, after centrifugation HDL-cholesterol was determined by cholesterol oxidase and peroxidase method [47]. LDL-cholesterol was calculated using Friedewald formula as following, LDL (mg/dl) = Total cholesterol –HDL-cholesterol- TAG/5 [48].

#### Assessment of cardiac troponin, inflammatory markers and anti-inflammatory marker

Assay of cTnI, TNF-α, IL-1β, PTX 3 and PON 1 were investigated in plasma samples and their plasma levels were determined using ELISA technique according to the manufacturer's instructions.

### Statistical analysis

The raw data were coded and transformed into coding sheets. The results were checked. Then, the data were entered into SPSS system files (SPSS package version 18) using personal computer. Output drafts were checked against the revised coded data for typing and spelling mistakes. Finally, analysis and interpretation of data were conducted.

The following statistical measures were used:

Descriptive statistics including frequency, mean, and standard deviation were used to describe different characteristics.

Kolmogorov – Smirnov test was used to examine the normality of data distribution.

Univariate analyses including: Mann Whitney test, Kruskal Wallis test, and Tamhane post Hoc test were used to test the significance of results of quantitative variables.

Diagnostic performance of CT, paraoxonase, interleukin 1-beta, pentraxin, TNF-α in diagnosing myocardial ischemia rats were assessed by sensitivity and specificity using ROC analysis.

The significance of the results was at the 5% level of significance.

## Abbreviations

CVD: Cardiovascular disease; CHD: Coronary heart diseases; n-3 PUFA: Omega 3 polyunsaturated fatty acids; cTnI: Cardiac troponin; TNF α: Tumour necrosis factor α; IL-1β: Interleukin-1 β; PTX 3: Pentraxin; ALA: Alpha linolenic acid; LDL: Low density liporprotein; HDL: High density lipoprotein; PON 1: Paraoxonase.

## Competing interests

The authors declare that they have no competing interests.

## Authors’ contributions

Dr Nounou, Dr Shalaby, and Dr Deif conceived the idea and designed the study. Dr Nounou and Dr Deif carried out the animal experiments. Dr Nounou and Dr Deif performed the statistical analysis and interpretation of the data. All the authors conducted equally the biochemical tests and provided critical corrections to the manuscript. All authors read and approved the final manuscript.
